# Histological Consequences of Needle-Nerve Contact following Nerve Stimulation in a Pig Model

**DOI:** 10.1155/2011/591851

**Published:** 2011-04-19

**Authors:** T. Steinfeldt, J. Graf, J. Schneider, W. Nimphius, E. Weihe, A. Borgeat, H. Wulf, T. Wiesmann

**Affiliations:** ^1^Department of Anaesthesiology and Intensive Care Therapy, University Hospital Giessen-Marburg, Faculty of Medicine, Philipps University Marburg, Campus Marburg, Baldingerstra*β*e, 35032 Marburg, Germany; ^2^Lufthansa AG, Aero Medical Center, Lufthansa Basis, Tor 21, 60546 Frankfurt am Main, Germany; ^3^Faculty of Medicine, Institute of Anatomy and Cell Biology, Philipps University Marburg, Robert-Koch Stra*β*e 8, 35032 Marburg, Germany; ^4^Department of Anaesthesia, Faculty of Medicine, Balgrist University Hospital, University of Zurich, Forchstra*β*e 340, 8008 Zurich, Switzerland

## Abstract

*Background*. Nerve stimulation can facilitate correct needle placement in peripheral regional anesthesia. The aim of this study was to determine whether the high threshold current is associated with reduced nerve injury due to fewer needle-nerve contacts compared with low current. *Methods*. In anaesthetized pigs, thirty-two nerves of the brachial plexus underwent needle placement at low (0.2 mA) or high current (1.0 mA). The occurrence of needle-nerve contact was recorded. After 48 hours, the nerves were analyzed for occurrence of histological changes. Nerve injury was scored ranging from 0 (no injury) to 4 (severe injury). *Results*. The frequency of needle-nerve contact was 94% at low compared to 6% at high current. The score was significantly higher at low (median [interquartile range] 2.0 [1.0-2.0]) compared to high current (0.0 [0.0-1.0] *P* = .001). *Conclusions*. Inflammatory responses were directly related to needle-nerve contacts. Hence, posttraumatic inflammation may be diminished using higher current for nerve localization.

## 1. Background

There is a reported incidence of 3% neurological deficits following peripheral regional blocks; however, most of these seem to regress without functional consequences within some weeks or months [1, 2]. Despite the introduction of insulated needles with short bevel and advances in needle guidance, such as electric nerve stimulation or ultrasound, the incidence of reported nerve injuries remained fairly constant [1]. 

Of note, causes for neurological deficits subsequent to regional anesthesia remain unknown most of the time. Among putative mechanism, needle-nerve contact leading to direct or indirect nerve injury may be involved. Only few data exist that evaluated causes and consequences of needle-nerve contact or nerve injury related to regional anesthesia [3–6]. Voelckel and coworkers recently reported inflammatory response of target nerves subsequent to needle placement for regional anesthesia in a pig model [7]. Here, marked inflammatory response of the peripheral nerve system was found, notably after needle placement with low current.

The aim of this study was to challenge the hypothesis that high threshold current (1.0 mA) is associated with a reduced likelihood of direct needle-nerve contact compared with low threshold current (0.2 mA), thereby reducing the potential for needle-related nerve injury. Primarily defined outcome variable was an established score representing the presence and magnitude of posttraumatic regional inflammation, occurrence of intraneural hematoma, and signs of myelin damage [6].

## 2. Methods

### 2.1. Animals

The experimental procedures were approved by local authorities (Regional board, Giessen, Hessen, Germany: VI63-19c20/15c MR 20/13-supplement 2006; Ref: 50/2009, Study no. 3), and the study was performed in compliance with the Helsinki convention for the use and care of animals. In this study, 7 female pigs (Deutsche Landrasse) weighing 29–42 kg (mean 34 kg) were used.

### 2.2. Anesthesia

Following premedication, general anesthesia was induced (propofol: 0.2 mg kg^−1^ min^−1^, IV; sufentanil: 0.5 *μ*g kg^−1^h^−1^, IV) as described recently [6, 8, 9]. All pigs were ventilated with pressure controlled ventilation (Siemens Servo 300; Maquet Critical Care, Darmstadt, Germany). The pigs were kept anesthetized for 48 hours. Adequate anesthesia was ascertained by adapting the dosage of propofol (maximum 0.3 mg kg^−1^ min^−1^) and sufentanil (maximum 1.5 *μ*g kg^−1^h^−1^). Fluid maintenance was with Ringer's lactate solution 3–5 mL kg^−1^h^−1^. The body temperature was assessed and kept constant using warming blankets (Bair Hugger Model 540; Arizant Healthcare Inc., Eden Prairie, USA).

### 2.3. Instruments

An insulated short bevel stimulation needle (Stimuplex A; B. Braun, Melsungen, Germany; 22-gauge; 30° bevel, 5 cm in length) connected to a nerve stimulator (Stimuplex HNS11, B. Braun, Melsungen, Germany) was applied for nerve stimulation. The device delivered a square wave current of 1.0 mA or 0.2 mA, the impulse duration was 0.1 ms with a frequency of 2 Hz. A neutral electrode was placed 30 cm from the site of needle insertion.

### 2.4. Surgical Preparation

**Figure 1:**
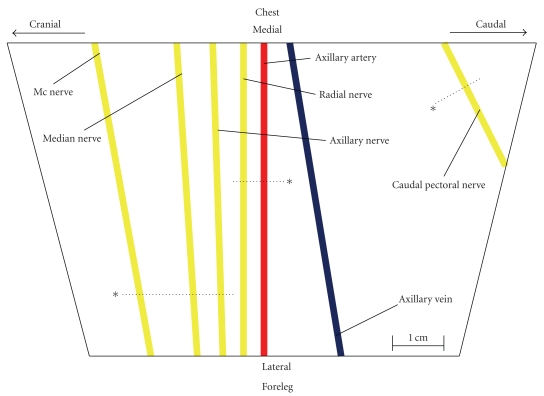
Schematic illustration of the right porcine brachial plexus. *, visual reference (suture); dotted line, imaginary line starting from the visual reference; mc nerve, musculocutaneous nerve. Musculocutaneous, median, radial and caudal pectoral nerve were selected for needle placement with low (0.2 mA) or high (1.0 mA) threshold current on the corresponding imaginary line. For detailed description please refer to Steinfeldt and coworkers [6].

The anesthetized animals were placed in the supine position with both fore limbs slightly abducted. Using an aseptic technique the axillary region was opened carefully by blunt dissection on both sides. The surgical approach was minimized to prevent bleeding related to dissection of muscular tissue or larger vessels. After cautious dissection of the vascular nerve sheath, the brachial plexus were exposed. Contacts by surgical instruments to nerves were prevented. Nerve connective tissue within the vascular nerve sheath was not removed. Subsequent to direct visibility of all plexus nerves, sutures serving as visual references were inserted intramuscularly (Figure 1). All nerves of the brachial plexus were identified, and anatomic landmarks as well as the localization of sutures were documented photographically. The anatomical landmarks of the undertaken approach were illustrated recently (Figure 1) [6]. 

Cefuroxim 80 mg kg^−1^d^−1^ IV was administered in all animals, and anesthesia was maintained as described before [6]. After 48 hours, the wound was reopened under general anesthesia, and the nerves of the brachial plexus were extracted. Photographs and visual references (sutures) guided the removal of treated nerve tissue. All animals were sacrificed at the end of the study period by an intravenous injection of potassium chloride (4 mmol kg^−1^).

### 2.5. Needle Placement and Control (Sham) Interventions

Altogether eight needle placements were scheduled for each animal with regard to the brachial plexus: four needle placements were applied using a 0.2 mA current intensity (low-current-treatment group), and another four needle placements utilizing 1.0 mA (high-current-treatment group). During all interventions, the current intensity used on each nerve was only known to the investigator (TW) in control, who was exclusively responsible for the selection of the nerve stimulator setting during needle placement. The operator undertaking the needle placement (TS) was blinded to the current output administered and followed the directives of the investigator in control manipulating the setting of the neurostimulator. Utilizing an internet-based randomization tool (http://www.randomizer.org/), the particular mode of treatment (needle placement with low current (0.2 mA) or high current (1.0 mA)) was allocated to all nerves just prior to the needle placement.

The musculocutaneous, the median, the radial, and the caudal pectoral nerve were selected for needle placement via neurostimulation. All needle placements were executed from cranial to caudal, beginning on the right hand side. The procedure was then continued on the left hand side, again from cranial to caudal. 

As a default setting, the needle was initially placed on nerve-connective tissue (muscle fascia and soft tissue) 5 mm lateral of the target nerve. The needle tip was then approaching the target nerve in an angle of 45° to 60°. During this approach, the nerve stimulator continuously delivered a predefined output current intensity of either 0.2 mA or 1.0 mA. The current intensity was not modified during needle positioning. The needle tip was pushed in 1 mm increments towards the target nerve until an adequate neuromuscular response (cloven hoof and lateral thorax) has been achieved. The final needle position was determined based on a minimal but specific neuromuscular response of the preselected target muscle. Subsequently, the needle tip to nerve distance was measured by means of a ruler. The distance was recorded in one millimeter increments. Direct contact of a needle tip with a nerve was documented. Thereafter, the stimulation needle was left in the final position for a period of 40 seconds with ongoing stimulation according to the pre-adjusted output current. All decisions, for example, whether or not an attempt has been accomplished successfully, and all assessments (i.e., measurement of distances and needle-nerve contact) were undertaken by the investigator in control. Following completion of the interventions, the opened tissue around the brachial plexus was carefully closed and sutured.

### 2.6. Control Groups

The left axillary nerve of each pig was the designated control group (nontreatment-brachial plexus); that is, this nerve was dissected but not exposed to any treatment at all. Thus, histological analysis of this nerve enabled us to determine whether the surgical approach itself had any influence on nerve integrity or associated inflammation. Additionally, the gluteal region was opened, and the left sciatic nerve was resected. The sciatic nerve represented a nerve tissue that was neither exposed to any potential surgical trauma (nontreatment-sciatic nerve), nor any needle placement treatment. Thus, all possibly confounding variables with respect to neuroinflammation such as systemic inflammation following needle placement, surgery, anesthesia, or any other intervention could have been detected. Ligature of a tibial nerve with subsequent histomorphological analysis was considered the control mimicking maximum nerve trauma.

Besides the surgical trauma, placement of the needle or the electric stimulation itself, irrespective of any mechanical alteration, may contribute to nerve injury or inflammation. To control for such current-related injuries, two animals with five nerves each were either exposed to (i) a current of 1.0 mA via direct needle-nerve contact for 40 sec, (ii) needle-nerve contact without application of electrical current, or (iii) close needle-nerve placement (4 mm distance) with application of 1.0 mA. To avoid muscle twitches potentially reducing the needle-nerve distance, a neuromuscular blocking drug (rocuronium, 1 mg kg^−1^) was given for the latter control group.

### 2.7. Histology

#### 2.7.1. Tissue Preparation

Each specimen (1–1.5 cm in length) was fixed by immersion in Bouin Hollande for 48 hours. After fixation, all tissue blocks were extensively washed in 70% 2-propanol and processed for paraffin embedding. Series of tissue slices (7 *μ*m) were taken throughout the specimen length.

#### 2.7.2. Histological Examination

Nerve specimens were cut, and every third slice was Giemsa stained. The initial histological analysis by light microscopy focused on the detection of the needle-nerve contact site (i.e., current nerve contact) which was usually characterized by circumscribed accumulation of inflammatory cells or structural damage (hematoma). Within the detected area, the pathologist searched for the most distinctive area of inflammatory response or the combination of inflammation and hematoma or myelin damage to locate the intervention site. Subsequently, at least four adjacent slices in both directions were alternately stained for either macrophages or myelin. Myelin was stained applying the technique by Kluver-Barrera to differentiate vital and avital myelin tissue [11, 12]. CD68 labelling [10] by immunohistochemistry was applied for the identification of macrophages and monocytes representing characteristic target cells with regard to neuroinflammation following nerve injury [13, 14]. As recently described, we developed a specific “injury score” (Table 1), adopting aspects from Hirata and coworkers [6, 15]. This score facilitates the characterization of the grade of inflammatory response (Giemsa staining), the occurrence of hematoma and the presence (or absence) of myelin damage (Table 1).

The relative number of CD68-positive monocytic cells (macrophages and monocytes) in relation to leucocytes was assessed by counting five representative visual fields including intravascular and extravascular areas (×200 magnification).

### 2.8. Statistical Analysis

The primary outcome measure was any nerve injury after needle placement utilizing low threshold current (0.2 mA) or high threshold current (1.0 mA) according to the grading of the “injury score” (Table 1). 

The sample size was chosen to provide a 90% power to detect a score value difference of 1.0 between the nontreatment group of the brachial plexus, the low current group, and high current group. A type-I error of 5% and a standard deviation of 0.5 in each group were assumed. Since most differences were expected between the nontreatment group of the brachial plexus and the current groups (0.2 mA, 1.0 mA), an unequal design with regard to the sample size and the allocation of the nontreatment group and the needle placement groups was executed. A specimen allocation of 1 : 3 : 3 was scheduled (nontreatment group of brachial plexus: high current : low current). The nontreatment sciatic nerve—that is, the control for systemic effects and confounders irrespective of any planned experimental intervention—has not been considered for the sample size calculation as well as the “current controls” and the positive control (nerve ligature). 

Using the PASS 2002 statistical package (Numbers Cruncher Statistical Systems, Kaysville, Utah, USA) a total number of at least 35 specimens was calculated. Considering a dropout rate of 20% (hematoma by nerve resection, complications during anesthesia, and accidental specimen destruction during laboratory processing), we planned five pigs to allow at least four intended needle tip placements per current group and animal. Two further pigs were scheduled for “current control” groups (needle-nerve contact with or without current, high current without nerve contact). 

Data are presented as median with 25th and 75th percentiles (interquartile range, IQR). Differences among the groups (low current (0.2 mA), high current (1.0 mA), nontreatment brachial plexus) regarding score value were determined by the Kruskal-Wallis test (i.e., global testing). A *P* value ≤.05 was selected as the criterion of significance. A confirmatory post hoc analysis including pairwise comparisons was applied in case of significant differences according to global testing (closed testing). For this, the Wilcoxon-Mann-Whitney test was selected. Statistics were performed using SPSS software for Windows (Release 15.0, SPSS, Chicago, IL). 

Only descriptive statistics have been applied with respect to the relative value of monocytic cells to leucocytes (mean ± SD) and needle-nerve distances (mm). However, a score value >1—that is, signs of inflammatory responses—was required for the assessment of monocytic cells.

## 3. Results

### 3.1. Animals

None of the 7 animals showed signs of local or systemic infection. Neither fever (>38°C) nor cardiopulmonary complications occurred throughout the experimental period.

### 3.2. Needle Placement and Immediate Macroscopic Evaluation

**Figure 2:**
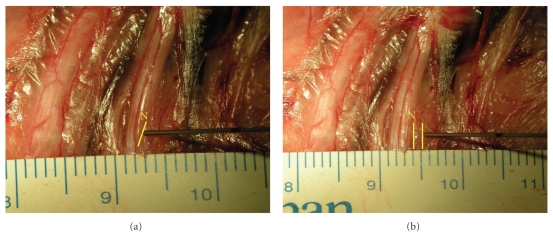
(a) Needle tip to nerve contact following needle placement with low threshold current (0.2 mA). The needle tip is located adjacent to nerve epineurium. (b) Distant needle placement with high threshold current (1.0 mA). A needle tip to nerve proximity of 2 mm was measured. N, radial nerve.

In the low current group (0.2 mA), direct needle-nerve contact was required in 15 out of 16 experiments (Figure 2) to elicit minimal twitches of the corresponding muscle. In contrast, in the high current group direct needle-nerve contact was rarely necessary (1 out of 15 cases) to induce a muscular response (Table 2). If needle-nerve contact was required, the needle had to be pushed slightly onto the nerve epineurium. Intraneural needle placement (i.e., nerve penetration) was not required to trigger muscular twitches. A metric evaluation (1 mm increments) of the needle-nerve distance revealed a considerably larger distance in the higher current threshold group (Figure 2) compared with the low current threshold group (Table 2). No (macroscopically) visible residuals were present after needle retraction.

### 3.3. Resected Nerve Specimens

Accidentally, eight nerves showed a distinctive hematoma, most likely caused during nerve resection (i.e., an iatrogenic lesion independent of the index intervention). Four nerves had undergone low current and four nerves high current stimulation, respectively. These nerves were excluded from further microscopic analysis.

### 3.4. Assessment of Nerve Injury Score

A variety of artifacts, that is, fascicle destruction, axonal damage in the absence of inflammatory cells, or avital myelin, were found in both the treatment and the control group. Intraneural hematoma with signs of myelin damage and increase of inflammatory cells was observed in the positive control (nerve ligature) only. Nerves with signs of regional inflammation revealed a remarkably high amount of monocytic cells among the leucocytes (Table 2).

### 3.5. Nerve Injury and Applied Stimulation Threshold Current

**Figure 3:**
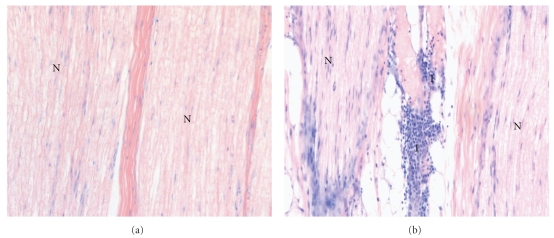
(a) Longitudinal microscopic view (×200, Giemsa stained) of the radial nerve after needle placement by means of nerve stimulation. A minimal threshold current of 1.0 mA was applied for needle positioning. The needle did not contact the nerve tissue. N, nerve fascicle; I, inflammatory cells. Score value, 0 (b) Longitudinal microscopic view (×200, Giemsa stained) of the median nerve after needle placement by means of nerve stimulation. A minimal threshold current of 0.2 mA was applied for needle positioning. The needle contacted the nerve tissue. N, nerve fascicle; I, inflammatory cells. Score value, 2.0.

**Figure 4:**
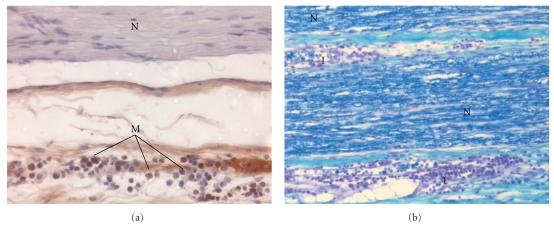
(a) Longitudinal microscopic view (×400, CD68 labeled [10]) of the median nerve after needle placement by means of nerve stimulation. N, nerve fascicle; M, brown, macrophages. Score value, 2.0. A minimal threshold current of 0.2 mA was applied for needle positioning. The needle contacted the nerve tissue. (b) Longitudinal microscopic view (×200, Kluver-Barrera [11, 12]) of the musculocutaneous nerve after needle placement by means of nerve stimulation. A minimal threshold current of 0.2 mA was applied for needle positioning. The needle contacted the nerve tissue. I, inflammatory cells; N, dark blue, myelinated vital nerve tissue. Score value, 2.0.

**Figure 5:**
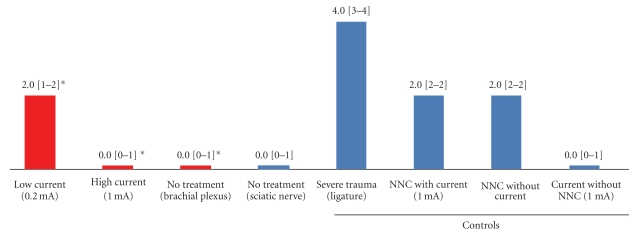
Treatment groups and controls. NNC, Needle-nerve contact; (), interquartile range; *, high current group was compared with low current and none-treatment (brachial plexus) group (Wilcoxon-Mann-Whitney test). The difference of applied nerve injury score values between high and low current treatment was significant (*P* < .01) whereas no significant difference (*P* = .46) was found between the nontreatment group (brachial plexus) and the high current treatment.

Corresponding to the primary outcome in the treatment groups, a difference was found (Figure 5): the median score value for nerve injury was higher after needle placement guided with low current (0.2 mA) compared to needle placement with high current threshold (2.0 IQR(1.0-2.0) versus 0.0 IQR(0.0-1.0) (Figures 3, 4, 5). The control group with direct needle-nerve contact revealed no differences with or without current (2.0 (2.0-2.0) versus 2.0 (2.0-2.0)) (Figure 5). 

A current intensity of 1 mA applied from a defined distance of 4 mm between needle and nerve did not reveal any signs of axonal injury, damage, or inflammation (Table 2, Figure 5). Herein, the pig was paralyzed to avoid any needle movement or needle-nerve contact.

Corresponding to global comparison (Kruskal-Wallis test) between high current, low current needle placement, and negative control (brachial plexus), a significant difference was found (*P* < .01). Hence, post hoc analysis was executed. Corresponding to the Wilcoxon-Mann-Whitney test without *P* adjustment, a significant difference (*P* < .01) between low and high current needle placement was observed, whereas no significant difference was found between no treatment brachial plexus and the needle placement with high current (*P* = .46).

## 4. Discussion

This study demonstrates (a) a dependency of threshold current and the frequency of needle-nerve contact during experimental regional anesthesia and (b) a pronounced regional inflammatory response subsequent to needle-nerve contact that was independent of the presence or absence of current. Interestingly, this posttraumatic inflammatory response was pronounced following needle placement applying low current (0.2 mA) compared to the application of high current threshold (1.0 mA). Regardless of the magnitude of the regional inflammatory response, neither intraneural needle location nor severe nerve injury—that is, structural nerve damage—was observed in either current threshold group. 

Recently, Voelckel and coworkers reported signs of inflammation following regional anesthesia in pigs with output currents <0.2 mA [7]. However, a number of methodological issues may limit the applicability of this data: first, the authors selected a time interval of six hours between electric nerve stimulation and nerve removal (i.e., dissection for further analysis). Of note, a pronounced inflammatory response may not occur earlier than 48 hours after nerve trauma [13, 14]. Second, a control group to allow validation of the applied methodology and evaluation of the results in the light of unknown confounders was missing. Third, the exact needle-nerve position subsequent to needle-nerve contact remained unclear: methylen-blue easily penetrates into all areas of the surrounding tissues therefore prohibiting an explicit (metric) assessment of the needle position in relation to the target nerve. Finally, macrophages, serving as important target cells representing posttraumatic inflammation, have not been considered for the description of inflammatory responses.

Our data are in line with the rationale that inflammatory cells accumulate as a response to any kind of nerve trauma [13, 14, 16]. Following nerve ligature mimicking maximum nerve trauma (positive control), massive leukocyte accumulation was found next to hematoma and myelin damage. In contrast, only mild signs of inflammation without structural damage in the nontreatment control were detected. The results of our control groups emphasize both the validity of the undertaken experimental intervention and the construct validity of the applied score [6]: a maximum trauma and a nontraumatic intervention were easily distinguishable and reproducible within the categories of the score. Between the nonintervention control of the brachial plexus and the nonintervention control of the sciatic nerve no differences were found. Therefore, it seems deducible that the surgical trauma (opening and closing of the axilla) did not interfere with the observed results with regard to the “treatment nerves”. Thus, relevant bias or unknown confounders may thus be neglected. 

The collected data concerning the relationship between needle tip to nerve proximity and electrical current intensity are in accordance with the perception that the level of output current corresponds to needle tip to nerve distance [17, 18]. Tsai and colleagues measured needle-nerve distances dependent on different currents in pigs as well [19]. However, they found a median needle-nerve distance of one millimeter at a minimal current of 1.0 mA, whereas we observed a distance of three millimeters when a minimal current of 1.0 mA was applied. In line with our findings, Tsai and coworkers reported needle-nerve contact in 95% of their experiments at a median current of 0.3 mA [19]. Whether the different anatomical site selected for the experimental setting, Tsai and coworkers used the sciatic nerve, may have contributed to the observed differences remains unknown [19].

Following needle-nerve contact, we found neither axonal nor myelin alterations but signs of regional inflammation. In most cases a gentle needle-nerve contact was sufficient to trigger minimal muscle twitches. In two cases, the nerves had to be stretched slightly during needle-nerve contact. Therefore, we assume that the aseptic inflammation is basically triggered by the needle tip comparable with, for example, a foreign particle reaction. Our control experiments with or without electric stimulation support the notion that inflammation induced via current only is very unlikely. 

In contrast to other investigators, we did not limit our assessment of regional inflammation to leucocytes but observed an increased relative number of macrophages and monocytic cells, respectively [7]. Therefore, we are confident that our findings represent a status that may well be termed “*posttraumatic*” inflammation.

According to our experimental setting, there is a paucity of data with regard to sound neurological followup after termination of anesthesia. Therefore, we are unable to relate the observed findings to any clinical manifestation or patient sequelae. Nevertheless, Eliav and coworkers demonstrated in rats that an aseptic inflammation of a peripheral nerve is indeed capable of provoking pain sensation that may be unrelated to apparent axonal damage [20]. 

The present investigation has a number of methodological limitations that need to be discussed. First, in our experimental setting, we utilized an “open brachial plexus model”. Although a percutaneous setup might have been desirable for a variety of reasons (e.g., closer to clinical practice, abdication of surgery, and no doubts with regard to conductance properties), a number of restrictions would have applied. Herein, the challenge to execute and subsequently identify the site of needle-nerve contact illustrates the most important obstacle for a controlled and reproducible experimental setting. 

We are confident that no relevant disturbance in electric stimulation and tissue or nerve-related conductance properties occurred for two reasons. (a) The target nerve remained embedded in the surrounding tissue (soft tissue, muscles, and fascia). Accordingly, the current is directed to the target nerve following the lowest impedance thereby passing through the surrounding tissue, which mimicks the clinically applied percutaneous approach. (b) The applied nerve stimulator provides a circuitry that generates a constant stimulating current despite eventual variability in tissue impedances. Therefore, the target nerve was expected to receive the adjusted current intensity irrespective of an open or closed model.

Second, with respect to the development of local inflammation, data from Mueller and others have demonstrated peak inflammation approximately 72 hours after nerve trauma [13, 14]. Thus, given our experimental setting with an observation period of 48 hours, we may have missed the peak of (post-)traumatic neuroinflammation. However, considering the experimental setting with indwelling catheters, long-term intubation, and mechanical ventilation, the risk for nosocomial infection in our pigs was not negligible. Such occurrence, that is, systemic inflammatory response syndrome, may have interfered with our analyses and was thus avoided. Again, Mueller and coworkers reported significant signs of inflammation following nerve trauma in almost all animals already 48 hours after the insult [13]. Weighing the risks and benefits, we felt comfortable with a setting allowing for insult-related inflammation without carrying a too high potential for nosocomial infection and have, therefore, limited the observation period to 48 hours.

Third, we lack any functional assessment of nerve integrity, for example, electromyography or postinterventional assessment of the animals. However, behavioural assessment is challenging, especially in larger animals such as pigs. In contrary, for rodents, for example, rats and mice, validated and reproducible instruments (i.e., hot plate test, incapacitance meter, von Frey electronic, etc.) are available [20, 21]. Therefore, functional assessment in pigs—as applied recently—should be interpreted with caution [22]. However, we focused on the assessment of morphological and pathophysiological reactions of nerves. Therefore, we used pigs within the applied experimental model, since nerve diameter, anatomic sites, and basic physiology are comparable with human beings, enabling the use of regional anesthesia equipment as applied in clinical routine.

Finally, heterogeneous aspects of different peripheral nerves (i.e., size and ratio of fascicles to connective tissue) could be associated with different needle positions during nerve stimulation with the same threshold current [19, 23].

Future experimental trials in smaller animals may thus, focus on the functional consequences of regional inflammation as described herein. It should be clarified whether regional neuroinflammation is associated with subsequent neurological deficits. Kiefer [16] and Moalem and Tracey [24] reported posttraumatic inflammation capable of inducing an impairment of neurological function, most likely due to the toxic mediators released by macrophages. Thus we cannot rule out that macrophage accumulation itself may lead to a neurological impairment independent from the applied trauma or duration of exposure.

## 5. Conclusions

We have demonstrated that needle-nerve contact in pigs does not cause axonal damage but may elicit a response denoted as aseptic inflammatory response. We are unable to draw any causal inferences with regard to functional consequences or clinical symptoms. Nevertheless, the present findings point out that needle-nerve contact following needle placement as applied in regional anesthesia may result in aseptic neuroinflammation.

## Figures and Tables

**Table 1:** Nerve injury score. Slight nerve damage is represented by the score grades 1 and 2. A severe nerve injury with structural damage is described with score grades 3 and 4. For detailed description with histological examples, please refer to Steinfeldt and coworkers [6].

Score value	Definition
0	No signs of neural injury or inflammation
1	Areas with slight accumulation of inflammatory cells
2	Areas with distinctive signs of inflammation
3	Areas with distinctive signs of inflammation* plus* haematoma
4	Areas with distinctive signs of inflammation *plus* myelin damage

**Table 2:** Treatment groups and controls. NNC, Needle-nerve contact; NN, needle-nerve; Giemsa, staining according to the Giemsa method; CD68, specific staining of CD68 positive leucocytes (macrophages) applying immunohistochemistry [10]; KB, myelin staining according to the Kluver-Barrera method [11, 12]; SD, standard deviation.

			Controls					
	High current (1.0 mA)	Low current (0.2 mA)	Non- treatment (brachial plexus)	Positive control (ligature)	Non- treatment (sciatic nerve)	Current (1.0 mA) without NNC	NNC with current (1.0mA)	NNC without current
Nerve specimen (*n*)	16	16	7	5	7	5	5	5
NNC (*n*)	1	15						
NN distance mean ± SD (mm)	2.9 ± 1.2	0.3 ± 1.0				4		
Slices (*n*) Giemsa/CD68/KB	2340/80/68	2490/92/71	780/16/16	400/52/48	850/16/16	870/25/45	780/52/48	890/82/94
Hematoma (Giemsa) (*n*, specimen)	0	0	0	5	0	0	0	0
Avital myelin (KB) (*n*, specimen)	0	0	0	5	0	0	0	0
Monocytic cells mean ± SD (%)	—	42	—	42	—	—	40	45
